# Manual therapy ameliorates neuromuscular dysfunction in spastic model rat: involvement of the C-Fiber-mediated CaMKII pathway

**DOI:** 10.3389/fnins.2026.1780013

**Published:** 2026-05-04

**Authors:** Yitian Lai, Hanbo Fan, Li Zeng, Lei Chen, Wu Li, Jiangshan Li, Heng Chen

**Affiliations:** 1 The Second Hospital of Hunan University of Chinese Medicine, Changsha, China; 2 College of Acupuncture-Moxibustion-Tuina and Rehabilitation, Hunan University of Chinese Medicine, Changsha, China; 3 Integrated Traditional Chinese and Western Medicine Hospital Affiliated with Hunan University of Chinese Medicine, Changsha, China

**Keywords:** cerebral palsy, C-fiber, manual therapy, spasticity, synaptic plasticity

## Abstract

**Background:**

This study investigated whether manual therapy applied to tendon organs ameliorated neuromuscular dysfunction in rats with spasticity induced by upper motor neuron injury associated with spastic cerebral palsy, and analyzed the potential involvement of the C-fiber-mediated CaMKII signaling pathway.

**Methods:**

Male rats were used to establish palsy models and divided into groups: Control, Model, Manual Therapy (MT), Capsaicin Treatment, Sham, CaMKII Inhibitor, and DMSO Solvent groups. Except for Control, all underwent pyramidal-tract destruction. After modeling, the MT group received manual therapy on the left-lower leg tendon organs. The Capsaicin group underwent sciatic nerve capsaicin treatment for C-fiber block on days 2 and 7; the Sham group had sciatic nerve exposure only. Both received daily manual therapy intervention for 14 days. The CaMKII Inhibitor and DMSO Solvent groups received intrathecal injections every 2 days (7 times total) without manual intervention. Spasticity-related behavioral indices, molecular expression, and neurotransmitter levels were assessed.

**Results:**

Manual therapy reduced the neurological deficit scores and muscle spasticity scores of model rats, improved the pathological morphology of the pyramidal tract and skeletal muscle, and regulated the expression of key molecules and neurotransmitters in the spinal cord and hippocampus. The therapeutic effects of manual therapy were significantly attenuated after C-fiber blockage, and although CaMKII inhibition could partially mimic the neuromodulatory effects of manual therapy, its efficacy in alleviating spasticity was inferior to that of manual-therapy intervention.

**Conclusion:**

Manual therapy appears to regulate CaMKII signaling via C-fiber afferent pathways to ameliorate neuromuscular dysfunction in a rat model of spasticity induced by pyramidal-tract lesion, thereby providing experimental evidence for the clinical application of optimized manual therapy parameters in the management of spasticity in patients with cerebral palsy.

## 1 Introduction

Spastic cerebral palsy (CP) is characterized by increased muscle tone and motor dysfunction, with core pathological mechanisms involving reduced inhibitory neurotransmitters in the spinal cord, pyramidal tract injury, and abnormal synaptic plasticity (Koman et al., 2004). Information transmission between neurons relies on synaptic function, and alteration of synaptic structure and function—referred to as synaptic plasticity—plays a critical role in neural integration. Recent studies have suggested that abnormal synaptic plasticity in CP may exacerbate motor dysfunction, although aberrant activation of hippocampal long-term potentiation (LTP) indicated that persistent imbalances in synaptic transmission efficiency may contribute to disease progression (Azeem et al., 2025). Calcium/calmodulin-dependent protein kinase II (CaMKII), a core regulator of LTP, directly influences the dynamic balance of synaptic plasticity through its phosphorylation state (Thapak et al., 2022), providing a potential target for therapeutic intervention.

Tendon organs, which are proprioceptors distributed at the muscle-tendon junction, dynamically monitor tension changes during muscle contraction, and play a key role in regulating muscle tone. In this study, we selected the tendon organs at the junction of the triceps surae and Achilles tendon in rats as the target site for manual intervention. Manual therapy (MT), a widely used complementary and alternative therapy, is believed to influence internal physiological states through external manipulation (He et al., 2020). In China, Tuina massage (a Chinese therapeutic technique) compression is a traditional manual therapy method that typically elicits a distinct “Deqi sensation”—a subjective experience of soreness and distension during compression (Chu et al., 2020). This sensation is considered an important physiological basis for the therapeutic effects of compression, and clinical studies have shown that Tuina massage is effective in treating muscle spasticity. It is important to note that the Deqi sensation induced by manual compression may be associated with C-fiber conduction (Chu et al., 2020; Jiang et al., 2016).

C-fibers, which transmit sensations such as slow pain, soreness, and distension, convert local mechanical signals from compression into sensory stimulation and transmit it to the central nervous system (CNS) to generate the Deqi sensation (Rudomin, 1990). Studies on manual therapy for analgesia have shown that it significantly reduces local tissue inflammation by decreasing inflammatory cytokine levels (Kauppila et al., 2017) and promotes the synthesis of myofibroblast proteins in muscles (Van Pelt Dw et al., 2019). Those findings suggested that manual therapy may represent a potential treatment option for spasticity in CP.

Our previous studies have demonstrated that spinal cord signal transduction related to tendon organ activation may involve CaMKII, which plays a potential role in regulating neural activities associated with muscle tone and influencing hippocampal LTP signaling cascades (Jiang et al., 2016; Chen, 2015). However, the anti-inflammatory and analgesic mechanisms underlying the Deqi sensation induced by manual therapy remain unclear. In the present study, we hypothesized that manual therapy improved spasticity through C-fiber-mediated modulation of CaMKII and subsequent regulation of inhibitory and excitatory neurotransmitter balance.

## 2 Materials and methods

### 2.1 Main reagents and instruments

Instruments: rat brain stereotaxic apparatus (Shanghai Yuyan Scientific Instrument Co., Ltd.), constant-speed propulsion syringe pump (Shanghai Yuyan Scientific Instrument Co., Ltd.), 25 μL microsyringe (Hamilton, Shanghai Hamilton), skull drill (Beijing Huachuangzhizao Technology Co., Ltd.), upright optical microscope (Nikon, Japan), JZL-III type soft tissue tension tester (Tianjin Mingtong Shiji Technology Co., Ltd.), SPECTRAMAX multi-function microplate reader (MDC, USA). Manual therapy instrument (Patent No. ZL202210366733.1, Self-developed device, Changsha, Hunan, China).

Reagents: Isoflurane (Shenzhen, RWD Life Science Co., Ltd), Pentobarbital sodium (Merck, Cat. No.: P11011), absolute ethanol (Sigma, USA, Cat. No.: 459836), capsaicin (GlpBio, USA, Cat. No.: N820430), calcium/calmodulin-dependent protein kinase II (CaMKII) inhibitor (KN-93, Cat. No.: GC35601), HE staining kit (Shanghai Biyuntian, Cat. No.: C0105S). Antibodies: N-methyl-D-aspartic acid receptor (NMDAR, Wuhan Boster, Cat. No: PA1058-1), Gamma-aminobutyric acid (GABA, Wuhan Boster, Cat. No: A07297-1), Glycine (Shanghai Xuan Ya Biology, product number: XY-KT-2082), enzyme-linked immunosorbent assay (ELISA) kits (Wuhan Cloud-clone): extracellular regulated protein kinase 1 (ERK1; Cat. No.: SEB357Ra, Batch No.: L240902669), adenylyl cyclase subtype 1 (ADCY1; Cat. No.: SEB348Ra, Batch No.: L240902694), CaMKII (Cat. No.: SEA655Ra, Batch No.: L240902694), *β*-hydroxybutyrate (β-HB, Cat. No: CEB022Ge, Batch No.: L240902675), lactic acid (Cat. No: CEV643Ge, Batch No.: L240902657).

### 2.2 Animal grouping and treatment

**Figure 1:**
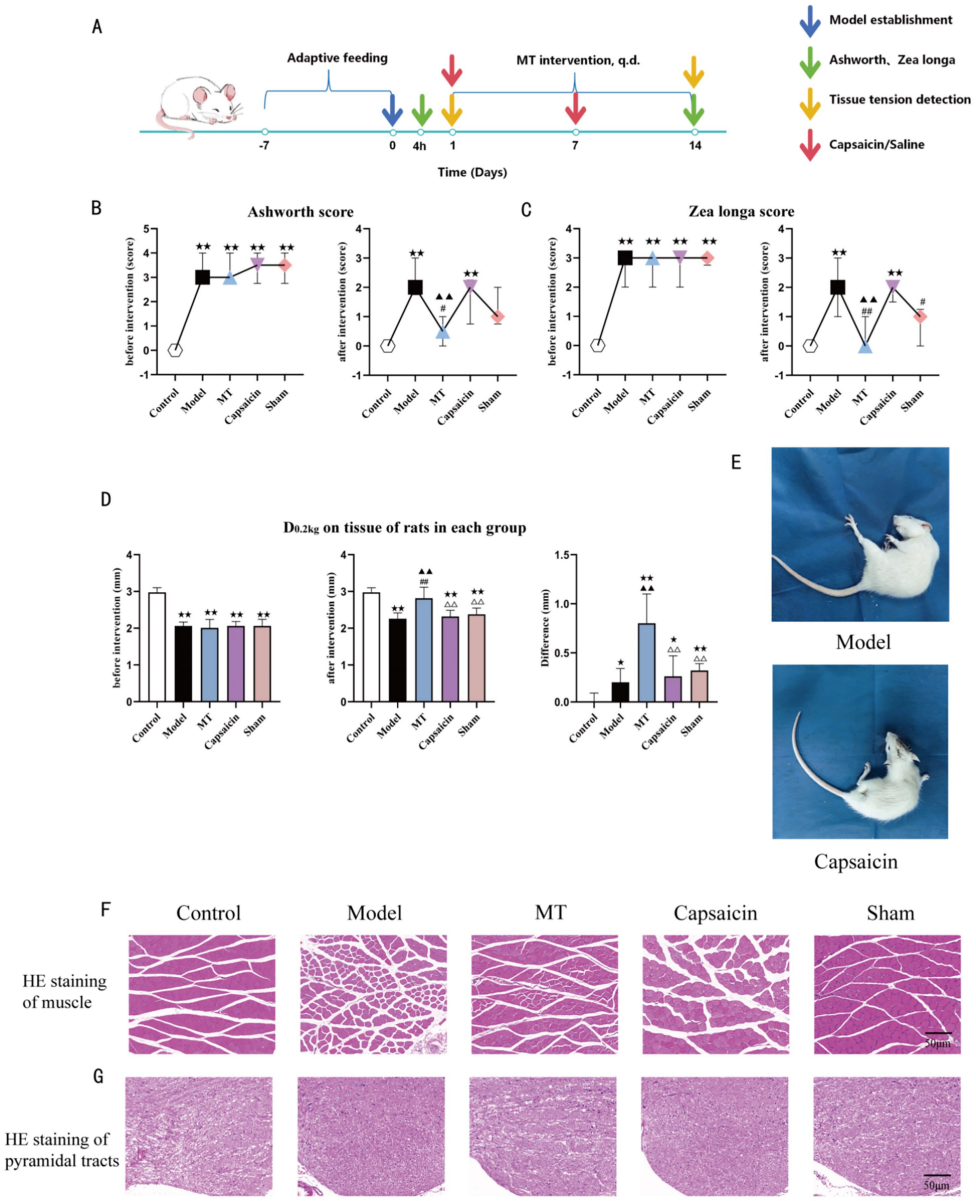
**(A)** Experimental flow chart; **(B)** Ashworth score (*n* = 10), expressed as M(Q25, Q75); **(C)** *Zea longa* score (*n* = 10), expressed as M(Q25, Q75); **(D)** Comparison of soft-tissue tone (*n* = 10), expressed as mean (SEM); **(E)** Condition of rat model establishment; **(F)** HE staining of the pyramidal tract in each group (bar = 50 μm, ×200). In the Model group, the nerve cells in the pyramidal tract showed loose structure, disordered arrangement, enlarged extracellular space, cerebral edema with enlarged brain cell volume, reduced nuclear volume, and increased staining intensity. Compared with the Model group, the MT, Capsaicin, and Sham groups showed certain improvements, but cellular swelling and small cystic spaces were still present, with no significant improvement in morphology. **(G)** HE staining of the quadriceps femoris in each group (bar = 50 μm, ×200). In the Model group, the myocyte spacing was increased and the muscle showed obvious atrophy; the MT group showed significant improvement; the myocytes in the Capsaicin group were still swollen; the Sham group showed significant recovery of myocyte spacing, but cellular swelling and inflammatory cell infiltration were still present. Compared with the Control group, ★★*p*<0.01, ★*p*<0.05; compared with the Model group, ▲▲*p*<0.01, ▲*p*<0.05; compared with the MT group, △△*p*<0.01; compared with the pre-intervention period, ##*p*<0.01.

**Figure 2:**
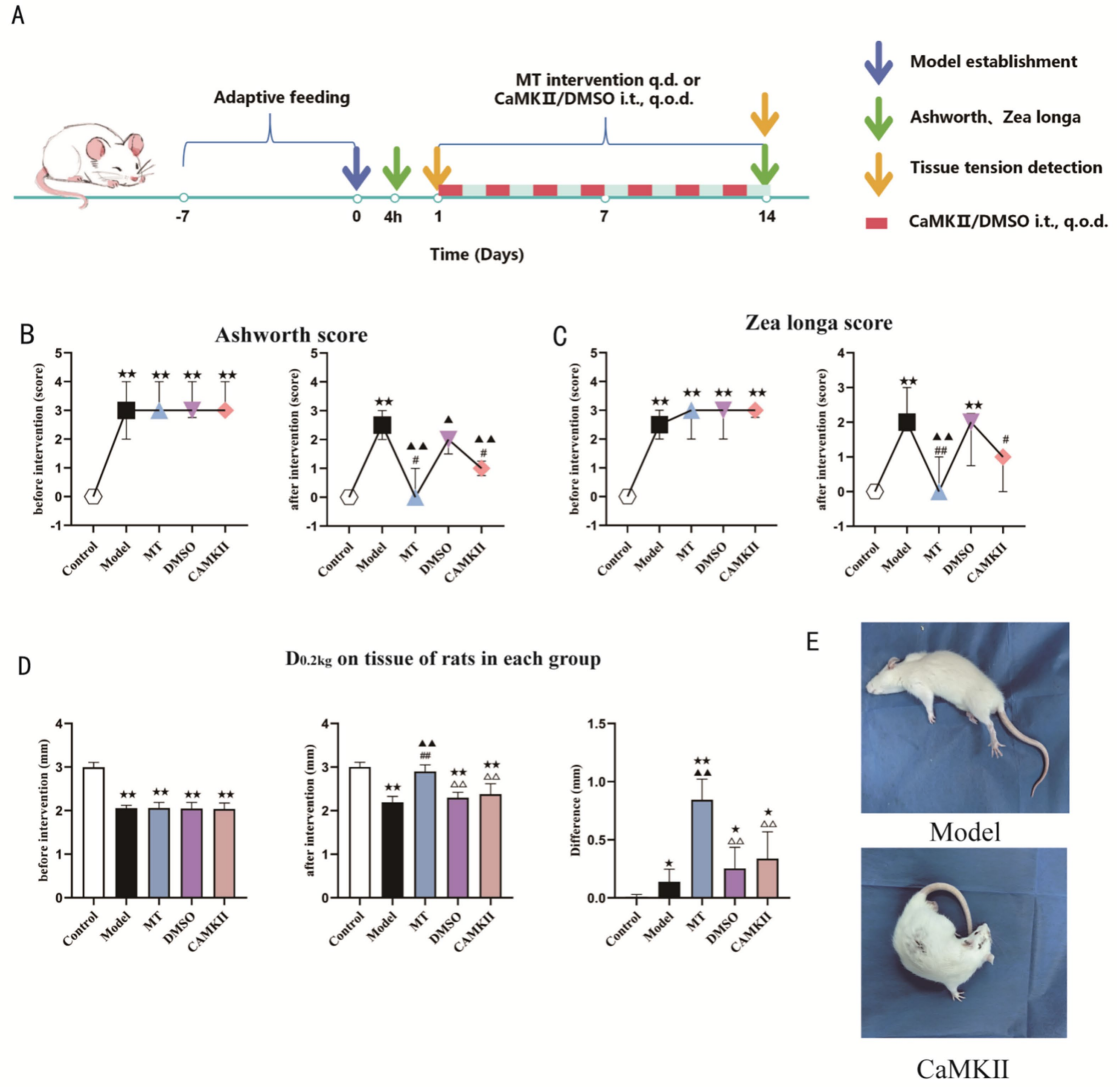
**(A)** Experimental flowchart. **(B)** Ashworth score (*n* = 10), M(Q25, Q75); **(C)** *Zea longa* score (*n* = 10), M(Q25, Q75); **(D)** Comparison of soft tissue tension (*n* = 10). Data are expressed as mean (SEM); **(E)** Model establishment status.

Male Sprague–Dawley rats weighing 200–240 g were purchased from Slack Jingda Laboratory Animal Co., Ltd. (Hunan, China) and reared in the Laboratory Animal Center of Hunan University of Chinese Medicine. All rats were housed under standard laboratory conditions (22 °C–27 °C, indoor humidity 50%–70%) with a 12-h light/dark cycle. Before the experiment, they were given free access to standard rat feed and water for 1 week, then grouped by the random number table method, with *n* = 10/gp. The detailed experimental procedure is shown in Figures 1A, 2A. All experimental procedures were conducted in accordance with the procedures approved by the Experimental Animal Ethics Committee of Hunan University of Chinese Medicine (Ethics Approval No.: HNUCM21-2405-05).

Groups (*n* = 10 each):

*Control* - Fed normally without any intervention.

*Model* - Established pyramidal tract lesion-induced spasticity model; after modeling, only inhalational anesthesia was administered for 14 days (consistent with each intervention group), without any intervention.

*MT (Manual Therapy group)* - Spasticity model was replicated; intervention was initiated on the 2nd day after successful modeling. After the rats were anesthetized by inhalation, a self-made Tuina manipulation stimulator (Jiang et al., 2021; Jiang et al., 2024) was used to press the tendon organs of the left leg of the rats, once a day, with 7 times as one course of treatment, and a total of 2 courses (14 times).

*Capsaicin group* - On the 2nd day after modeling, the left sciatic nerve of the rats was surgically isolated, wrapped for 24 h with a 1 cm^2^ wet gauze containing 2% capsaicin (Zhu et al., 2004), then sutured. The same capsaicin treatment was performed again on the 7th day. During this period, manual intervention was continuously performed for 14 consecutive days.

*Sham* - control for capsaicin treatment. On the 2nd day after modeling, the left sciatic nerve was wrapped for 24 h with a 1 cm^2^ wet gauze soaked in normal saline, then sutured. On the 7th day, the same procedure as the capsaicin treatment was repeated (capsaicin was replaced with normal saline). During this period, manual intervention was continuously performed for 14 consecutive days.

*CaMKII inhibitor* - starting from the 2nd day after successful modeling, intrathecal injection of CaMKII inhibitor was administered once every 2 days, a total of 7 times, without manual intervention.

*DMSO solvent* - vehicle control for CaMKII inhibitor. Starting from the 2nd day after successful modeling, intrathecal injection of DMSO solvent, as a vehicle control for the CaMKII inhibitor, was administered once every 2 days, a total of 7 times, without manual intervention.

### 2.3 Establishment and evaluation of the model

To simulate the spastic symptoms caused by upper motor neuron injury in spastic CP, we established a spasticity model by pyramidal tract destruction with absolute ethanol, referring to the classic method reported in previous studies (Yu et al., 2013). Rats were anesthetized with an intraperitoneal injection of 1% sodium pentobarbital (40 mg/kg), then fixed in a supine position. After hair removal and disinfection, the anesthetized rats were fixed in a stereotaxic apparatus. A midline incision of approximately 3 cm was made on the posterior scalp, and the skin, subcutaneous tissue, deep fascia, and periosteum were incised layer by layer to fully expose the bregma and sagittal suture. Stereotaxic injection was made at the following coordinates relative to bregma: anterior–posterior (AP), 10.0 mm; medio-lateral (ML), 0.8 mm; dorso-ventral (DV), 9.7 mm below the surface of the dura. These all-used coordinated from the atlases of Paxinos and Watson. A hole was drilled (approximately 0.7 mm in diameter) in the skull. Then, 15 μL of absolute ethanol was injected at a rate of 1 μL/min. After the injection, the syringe was kept in place for 5 min, then slowly withdrawn. After sufficient hemostasis, the skull hole was immediately sealed with bone wax, and the incision was sutured.

At 4 h after the rats regained consciousness, a Zea Longa neurological deficit score was obtained. Rats with a neurological deficit score of 1–3 points and a modified Ashworth spasticity score of 1–4 points were considered successfully modeled.

### 2.4 Manual-manipulation method

A self-made manual therapy stimulator was used as a manual-manipulation instrument, and the stimulation parameters were set according to previous experiments (Jiang et al., 2021; Jiang et al., 2024). Intervention was initiated on the 1st day after successful modeling: the tendon organs of the left leg of the rats were pressed with the optimal stimulation parameters (force: 0.7 kg, duration: 15 min, frequency: 10/min) (Jiang et al., 2016), perpendicular to the skin, once a day, with 7 times as one course of treatment, and a total of 2 courses (14 times). The force, duration, and stimulation mode of the manipulation were the main factors affecting the effect.

### 2.5 Capsaicin treatment

For the Capsaicin group, on the 2nd day after modeling the rats were anesthetized with 4% isoflurane (maintained at a concentration of 2.5%). The left sciatic nerve of the rats was surgically isolated and wrapped for 24 h with a 1 cm^2^, wet, gauze pad containing 2% capsaicin (Zhu et al., 2004). After removing the gauze, the incision was sutured. The same capsaicin, wet, compress operation was performed again on the 7th day. During the intervention period, manual therapy was repeatedly performed for 14 days. For the Sham group, the same surgical procedure was performed, but the capsaicin-saturated gauze pad was replaced with a normal saline-soaked gauze pad, but the other operations were the same.

### 2.6 Intrathecal injection

Starting on the 2nd day after successful modeling, the rats were given intrathecal injections of the specific CaMKII inhibitor, KN-93 (Jiang et al., 2015; Zhang et al., 2022). The hair on the rat’s dorsal waist was removed, and the skin was disinfected with povidone-iodine. The rats were anesthetized with 4% isoflurane (maintained at a concentration of 2.5%). During surgery, the rat’s iliac crest was gently held, and a 20 μL microsyringe equipped with a 30-ga needle was inserted vertically into the L4-L5 intervertebral space. Tail tremor or flicking indicated successful puncture. According to the instructions, a 100 μmol/L solution was prepared, and 15 μL was injected each time. Injections were administered once every 2 days, starting from the first day after successful model establishment, for a total of 7 times. The DMSO group received a surgical incision and an equal volume of DMSO via intrathecal injection, with the other operations being the same.

### 2.7 Evaluation of limb spasticity using the modified Ashworth scale

Limb spasticity of rats in each group was evaluated on the day after modeling and on the 14th day after intervention. Rats with a score on the Modified Ashworth Scale of 1–3 points on the day after modeling were included in the experiment, and those with a score of ≥4 points were excluded (see Figures 1A, green arrow). The scores were assigned as follows: Grade 0 = 0 point; Grade 1 = 1 point; Grade 1+ = 2 points; Grade 2 = 3 points; Grade 3 = 4 points; Grade 4 = 5 points. Lower scores indicated lower spasticity levels (Baunsgaard et al., 2016).

### 2.8 Evaluation of neurological deficits using the Zea Longa Scale

The neurological-deficit status of rats in each group was evaluated on the day after modeling and on the 14th day after intervention. Rats with a score on the Zea Longa Scale of 1–3 points after modeling were included in the experiment, whereas those with a score of 4 points were excluded (see Figures 1A, yellow arrow) (Longa et al., 1989).

### 2.9 Measurement of soft-tissue tension

The soft-tissue tension of rats in each group was measured on the day after modeling and the 14th day after intervention (see Figure 1A, yellow arrow). The rat’s head was fixed with a hood to keep it in a quiet state, and the soft-tissue-tension tester was connected, debugged, and calibrated. The hair on the left hind limb of the rat was shaved, and the muscle-tension band on the medial thigh of the quadriceps femoris was identified by palpation. The detection head was aligned with the detection area, and force was applied vertically and evenly for approximately 3 s, then slowly and evenly removed. The detection system automatically recorded the force-displacement curve during loading and unloading for data analysis.

Referring to the instrument manual and the previous research of our team (Jiang et al., 2024), the displacement corresponding to 0.2 kg force (D0.2kg) in the curve was used as the observation index, which reflected the soft-tissue tension of rat muscles. Each rat was tested 3 times with 10 min between tests, and the average value was taken as the actual value for the rat. The D0.2kg value is negatively correlated with soft-tissue tension (i.e., the smaller the D0.2kg value, the greater the soft-tissue tension). Measurements were performed once before and once after the intervention.

### 2.10 Histological examination of the pyramidal tract

Rats were anesthetized with an i.p. injection of 1% pentobarbital sodium at a dose of 40 mg/kg for tissue-sample collection: the whole brains of 3 rats in each group were fixed with 4% paraformaldehyde solution, then subjected to dewaxing, dehydration, paraffin embedding, sectioning (5 μm thick), and HE staining to prepare sealed sections of brain tissue for histological analysis. Under a light microscope, the damage (such as degeneration and necrosis) of pyramidal tract neurons was assessed and compared, and images were collected.

### 2.11 Histological examination of muscles

The quadriceps femoris tissue of the rats was fixed with 4% paraformaldehyde solution, then subjected to dewaxing, dehydration, paraffin embedding, sectioning (5 μm thick), and HE staining to prepare sealed sections of muscle tissue for histological analysis. Under a light microscope, the damage (such as degeneration and edema) of muscle cells was assessed and compared, and images were collected.

### 2.12 Immunohistochemical analysis

For each group, 3 brain tissue samples and 3 spinal cord tissue samples fixed with 4% paraformaldehyde solution were dehydrated, paraffin-embedded, and sectioned (approximately 4 μm thick) to observe the expression of glycine, NMDAR, and GABA in the hippocampus, and the expression of NMDAR and GABA in the spinal cord. The sections were dewaxed with xylene, treated with gradient ethanol, and rinsed with water. Antigen retrieval was performed using sodium citrate solution and a microwave oven, followed by blocking with 10% normal goat serum. The sections were then sequentially incubated with the diluted primary antibody (1,400) at 37 °C for 2 h, an appropriate amount of reaction-enhancement solution was added, and the samples were incubated at room temperature for 20 min. Then, an appropriate amount of enhanced enzyme-labeled goat anti-rabbit IgG polymer (1,100) was added, and the samples were incubated at room temperature. Subsequently, the sections were sequentially stained with DAB chromogenic solution and hematoxylin solution for 30 min, and the reaction time was controlled under a microscope. The Eclipse Ci-L imaging microscope was used to select the target area of the tissue for high-magnification imaging. After imaging, Image-Pro Plus 6.0 analysis software was used, with pixel area (pixel) as the standard unit, to measure the cumulative optical density (OD) of positive signals in 3 visual fields of each section and the corresponding positive pixel area (Area) (brown color indicated positive staining). The average optical density (AOD value) was calculated as follows: AOD value = cumulative optical density value/positive pixel area.

### 2.13 Elisa

The L1-L2 segments of the spinal cord of 7 rats in each group were collected and fully homogenized and lysed. The cell lysate was centrifuged at 12000 g for 5 min at 4 °C, and the supernatant was used for protein quantification using a bicinchoninic acid (BCA) protein concentration assay kit (Biyuntian). The levels of CaMKII, ERK, and ADCY1 in the spinal cord were assessed using ELISA kits according to the manufacturer’s instructions, with duplicate wells for each sample. The hippocampal tissue of 7 rats in each group was also fully homogenized and lysed using the same procedure, and the levels of *β*-HB and lactic acid in the hippocampus were assessed.

### 2.14 Statistical analysis

SPSS 25.0 software was used for statistical analysis. In this study, randomization was performed using a random-number table. For the measurement data, normality and homogeneity of variance tests were first conducted. If the data satisfied normality, they were expressed as mean (standard deviation, SD), and one-way analysis of variance (One-Way ANOVA) was used for pairwise comparisons between multiple groups. The LSD method was used when variances were homogeneous, and the Dunnett T3 method was used when variances were heterogeneous. For data not meeting normality, data were described as M (Q25, Q75), and non-parametric tests were used. A *p*-value ≤ 0.05 was considered statistically significant. For graphical presentation, data were shown as mean ± standard error of the mean (SEM).

## 3 Results

### 3.1 Changes in muscle strength were closely related to pyramidal tract injury

Pyramidal tract injury and changes in muscle strength of rats are the core pathological links of CP-related spasticity in this study. The results showed that in the Model group, the pyramidal tract area exhibited cerebral edema with enlarged brain cell volume, along with reduced nuclear volume, increased staining intensity, enlarged extracellular space, and disordered cell arrangement. Compared with the Model group, the MT, Capsaicin, and Sham groups showed certain improvements; however, cellular swelling and formation of small cystic spaces were still observed, with no significant improvement in cell morphology (see Figure 1G).

Spastic CP is characterized by motor control disorders centered on hyperactive stretch reflexes, which lead to decreased core muscle strength. This further causes impairment in force transmission and control, resulting in balance disorders and central-peripheral coordination disorders, and ultimately restricts the development of motor function (Valadão et al., 2025). The results of the present study showed that the neurological deficit scores and muscle spasticity scores of rats in the Model group significantly increased (see Figures 1B,C, 2B,C). The rats exhibited behaviors such as rotating in place and inability to move straight forward, and the affected leg showed obvious rigidity (see Figures 1E, 2E). After the completion of compression intervention, all these symptoms were significantly reduced.

The results of this study showed that before intervention, the D0.2kg values of rats in the Model, MT, Capsaicin, and Sham groups were all lower than those of the Control group, with no statistically significant differences among the groups. This indicated successful model establishment; the muscle tone of all model groups was increased. After intervention, compared with the Model group, the D0.2kg values of the MT group, Capsaicin group, and Sham group were higher. The D0.2kg values of the MT group and Sham group were higher than they were in the pre-intervention period. For the pre-intervention and post-intervention difference values: compared with the Control group, the D0.2kg difference values of the MT group, Capsaicin group, and Sham group were increased; compared with the Model group, the D0.2kg difference values of the MT group, Capsaicin group, and Sham group were also increased (see Figure 1D). The above results suggest that the soft tissue tone of the hemiplegic side in rats increased after model establishment, and manual stimulation can reduce soft tissue tone.

The results showed that the muscle tone of the quadriceps femoris in the palsy-model rats was significantly increased. Histological analysis revealed disordered myofiber arrangement and loose muscle tissue structure, which contrasted with the dense arrangement of normal muscle tissue; inflammatory cell infiltration was observed between muscle fibers. In addition, the rats showed obvious muscle atrophy: in the atrophic muscle tissue, there was reduced myofiber diameter, significantly decreased cross-sectional area, myofibers tending to be round in shape, and inflammatory cell infiltration. After Tuina intervention, the soft-tissue tone of the affected-side quadriceps femoris decreased significantly; muscle tissue staining showed that myocyte morphology tended to be normal, and inflammatory infiltration was reduced (see Figure 1F).

### 3.2 Capsaicin treatment and manual-therapy effects

In this study, 2% capsaicin was used for defunctionalization of C-fibers in the rat sciatic nerve (Zhu et al., 2004), in order to observe whether mechanical compression exerted its effect by transmitting signals to the spinal cord via C-fibers, thereby improving the muscle spasticity state of rats. The results showed that after manual intervention on the tendon organs of the left lower limb in palsy rats, the ipsilateral soft-tissue tone decreased and the spastic state was significantly alleviated. This suggested that the therapeutic effect of Tuina manipulation on spastic rats may have been related to C-fiber-mediated effects.

**Figure 3:**
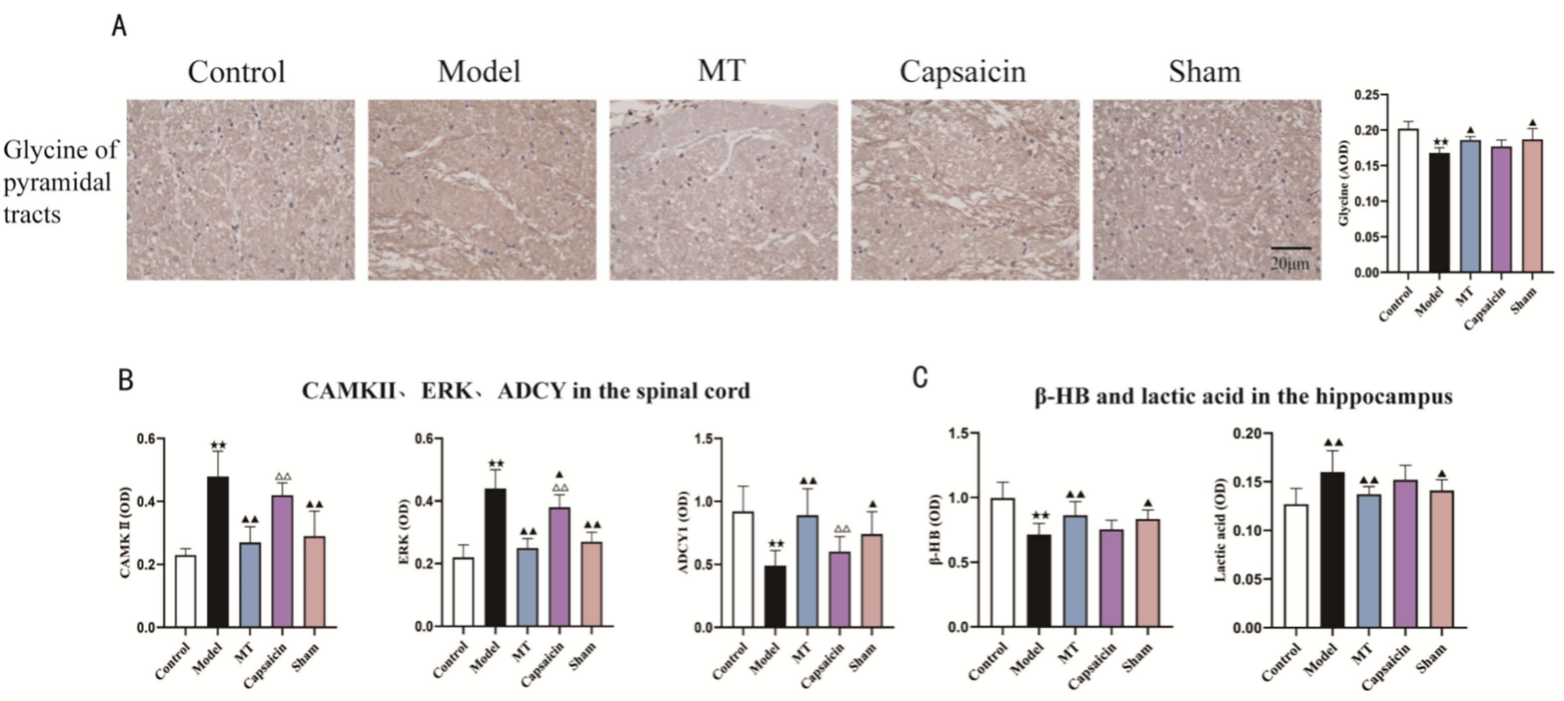
(A) Immunohistochemical expression of glycine in the hippocampus of rats in each group (bar = 20 μm, ×400) and statistical bar chart; **(B)** Expression levels of CaMKII, ERK, and ADCY1 in the spinal cord of rats in each group (*n* = 7); **(C)** Expression levels of β-hydroxybutyrate (β-HB) and lactic acid in the hippocampus of rats in each group (*n* = 7). Data are expressed as mean (SEM). Compared with the Control group, ★★*p* < 0.01, ★*p* < 0.05; compared with the Model group, ▲▲*p* < 0.01, ▲*p* < 0.05; compared with the MT group, △△*p* < 0.01; compared with the pre-intervention period, ##*p* < 0.01.

The results of this study showed that in the model rats, the levels of CaMKII and ERK in the spinal cord were significantly elevated, whereas the level of ADCY1 was markedly reduced. After manual therapy intervention, the levels of CaMKII, ERK, and ADCY1 in the spinal cord of rats all changed significantly, as shown in Figure 3B. In addition, the immunohistochemical results indicated that the glycine content in the pyramidal tract was lower in the model rats, whereas a large amount of the inhibitory neurotransmitter glycine was released after manual therapy intervention, as shown in Figure 3A. In addition, in the model rats the level of *β*-HB in the hippocampus was significantly lower and the lactic acid content was higher. After manual therapy intervention, the β-HB level was significantly higher and the lactic acid content was markedly lower, as shown in Figure 3C.

### 3.3 CaMKII reduced NMDAR content and increased the level of GABA

CaMKII is a key signal-transduction molecule in the CNS. Therefore, in the second part of this study, CaMKII inhibitor was used for intervention to investigate whether the effect of manual therapy on C fibers was closely related to the key factor CaMKII. Studies have shown that in neuropathic hyperalgesia, CaMKII inhibitors can significantly alleviate hyperalgesia, suggesting that CaMKII is a downstream key molecule in C-fiber-mediated pain However, the measurement of soft-tissue tension showed that the therapeutic effect of the MT group was significantly better than that of the CaMKII inhibitor group, which may have been related to the direct action of mechanical manual therapy on the ipsilateral tendon organs of the rats. See Figure 2D for details.

**Figure 4:**
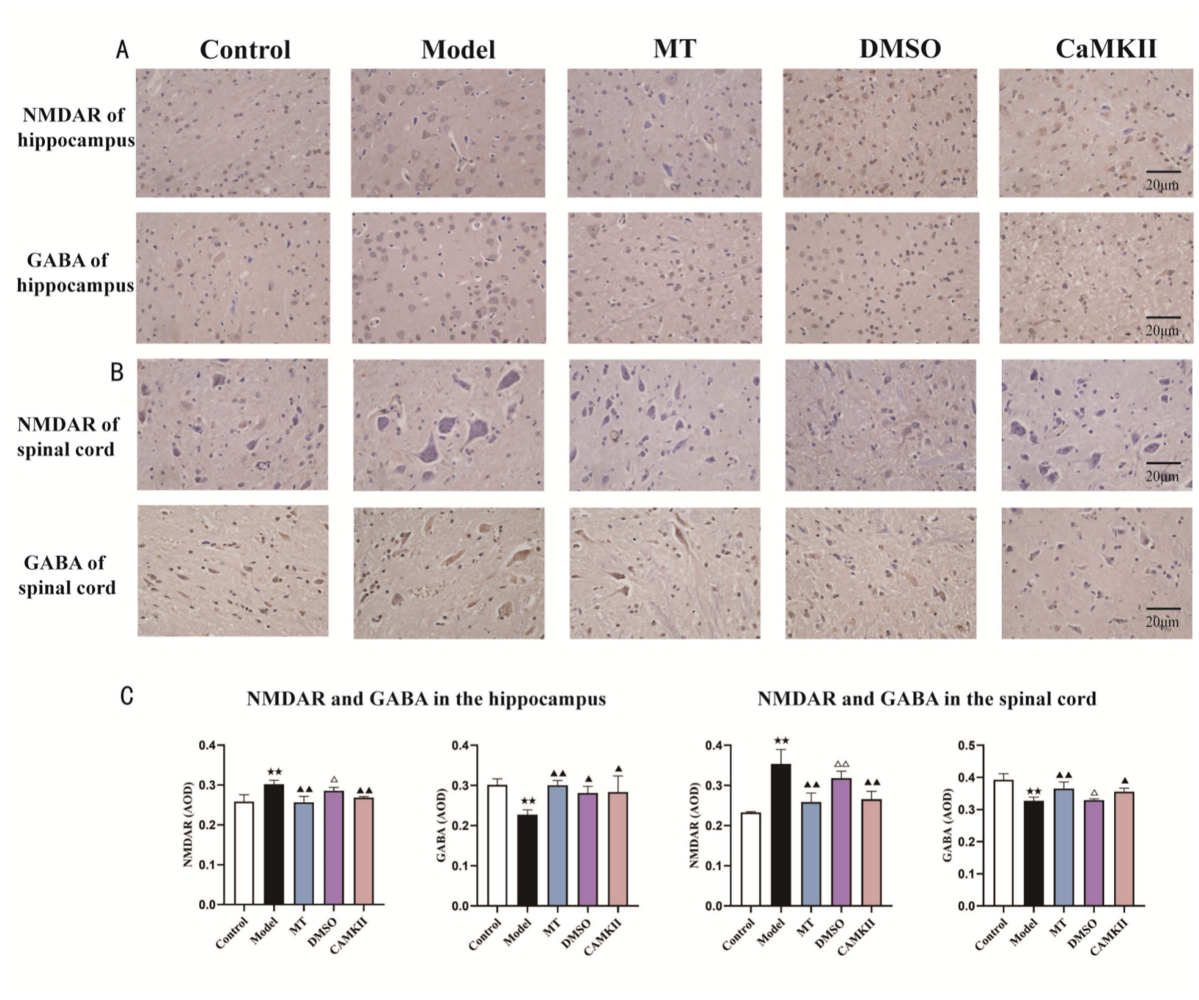
**(A)** Immunohistochemistry of NMDAR and GABA in the hippocampus of rats in each group (scale bar = 20 μm, 400× magnification); **(B)** Immunohistochemistry of NMDAR and GABA in the spinal cord of rats in each group (scale bar = 20 μm, 400×); **(C)** Statistical analysis of NMDAR and GABA contents in the hippocampus and spinal cord of rats in each group by immunohistochemistry (*n* = 3). Data are expressed as mean (SEM). Compared with the control group: ★★*p* < 0.01, ★*p* < 0.05; compared with the model group: ▲▲*p* < 0.01, ▲*p* < 0.05; compared with the MT group: △△*p* < 0.01, △*p* < 0.05.

After manual intervention on tendon organs in spastic model rats, the concentration of NMDAR in the spinal cord and hippocampus was significantly higher (p), whereas the concentration of GABA was significantly lower. After intervention with the CaMKII inhibitor, the GABA content in the brain and spinal cord increased, and the effect was similar to that of manual therapy. See Figures 4A–C for details.

## 4 Discussion

In clinical studies (Noble et al., 2019), patients with spastic CP experienced loss of descending excitatory and inhibitory signals, abnormal input of spinal motor neuron signals, and subsequent changes in muscle structure and function (including increased muscle tone, motor dysfunction, and spasticity) due to non-progressive brain injury. Lesions of the pyramidal system in CP lead to the loss of various types of inhibitory functions (Steinbok, 2007); injury to the supraspinal centers results in the development of spastic CP (Boychuck et al., 2020). The spinal cord and cerebral pyramidal tract are critical components of motor control, and spasticity may arise from abnormal synaptic potentiation in the motor cortex-spinal cord pathway, which is closely associated with CaMKII (Zou et al., 2025).

Numerous clinical studies have demonstrated that the quadriceps femoris exhibits the most severe spasticity in CP (González-Matilla et al., 2025); therefore, the quadriceps femoris was selected as the target effector muscle group for detection in this study. Tendon organs, as muscle-tone receptors distributed at the musculotendinous junctions, can activate inhibitory interneurons (Kanning et al., 2010) to suppress excessive stretch reflexes. Stretch receptors within muscle spindles and tendon organs transmit impulses via afferent nerves to excite *α*-motor neurons in the anterior horn of the spinal cord (Rudomin, 1990), and then regulate the coordinated movement of synergistic and antagonistic muscles through reflex efferent branches. Therefore, in this study, manual compression was applied to the tendon organs at the junction of the left triceps surae and Achilles tendon in rats to observe its therapeutic effects in alleviating muscle spasticity and increased muscle tone induced by CP.

C-fiber nociceptors can respond to mechanical stimulation induced by manual therapy, chemical stimulation from capsaicin, and other such stimuli. High-dose capsaicin can induce defunctionalization of capsaicin-sensitive chemical nociceptors on C-fibers (Zhu et al., 2004; Abdel-Salam and Mózsik, 2023). In this study, a sham operation group was included as a control to rule out local stimulation of the sciatic nerve caused by surgical manipulation and gauze wrapping, and to control for confounding factors related to mild local inflammation potentially induced by the capsaicin. Additionally, studies have demonstrated that electrical stimulation of C-fibers in the sciatic nerve allows for the recording of C-fiber-evoked field potentials in the superficial layer of spinal cord neurons in rats, indicating that C-fiber signals are directly involved in the regulation of spinal cord neuronal networks (Abdel-Salam and Mózsik, 2023).

In the present study, the levels of CaMKII, ADCY1, and ERK in the spinal cord of rats with the spasticity model were all significantly altered. The expression and activity of CaMKII directly affect synaptic strength and neuronal excitability, playing a pivotal role in neural signal transmission and synaptic plasticity (Hell, 2014). ADCY1, in turn, is a key molecular regulator of synaptic plasticity in the anterior cingulate cortex (Bliss et al., 2016) and also exerts a critical role in the modulation of long-term potentiation (LTP) in the spinal dorsal horn (Wei et al., 2006). The ERK family is central to synaptic plasticity, and the role of ERK in LTP of C-fiber-evoked field potentials in the spinal dorsal horn is associated with pathological pain (Xin et al., 2006).

Our findings revealed a significant decrease in the level of *β*-HB in the hippocampus of model rats. *β*-HB can attenuate injury to isolated hippocampal neurons in rat models of status epilepticus and improve cognitive function by enhancing synaptic plasticity and adult hippocampal neurogenesis (Landgrave-Gómez et al., 2015). Furthermore, the lactic acid level in the hippocampus of model rats was significantly elevated. Studies have shown that lactic acid is markedly increased in animal models of acute hypoxic–ischemic brain injury, with the magnitude of the increase positively correlated with infarct volume and neurological deficit scores (Overgaard et al., 2012). When cerebral blood flow is reduced, cerebral lactic acid accumulation can induce intracellular acidosis, exacerbating blood–brain-barrier disruption and neuronal apoptosis (Hladky and Barrand, 2024).

The role of glutamate in synaptic plasticity in rats with CP is primarily mediated by its receptor-dependent signaling pathways. Excessive activation of NMDAR may induce neuronal excitotoxicity, leading to neuronal damage and dysfunction (Neame et al., 2019). In the present study, the levels of NMDAR in the spinal cord and hippocampus of model rats were significantly elevated. Enhanced NMDAR activity can promote the induction of hippocampal LTP and trigger synaptic morphological changes, which may subsequently alter the structural and functional properties of synaptic connections between inhibitory interneurons and pyramidal neurons (Udakis et al., 2020), thereby attenuating the therapeutic efficacy of tendon organs in relieving muscle spasticity. In contrast, the levels of GABA were markedly decreased in model rats, which may be attributed to the abnormal activation of CaMKII, thereby impairing the function of GABAergic neurons through multi-level cascade reactions (Arora et al., 2024). At the spinal cord level, GABA is primarily released by Renshaw cells, which coordinate the synergistic contraction of antagonistic muscle groups by inhibiting *α*-motor neurons; under pathological conditions, GABA interacts with microglia to participate in the regulation of neuroinflammation (Udakis et al., 2020). Studies have demonstrated that CaMKII overexpression results in defects in the aggregation of synaptic scaffold proteins in GABAergic neurons (Hosokawa et al., 2021); electron microscopy has revealed a significant reduction in the density of GABA receptors on the postsynaptic membrane, and sustained activation of CaMKII induces apoptosis of GABAergic neurons. In the present study, after manual therapy, GABA levels increased significantly, which may have been due to mechanical stress generated by the compression stimulation of tendon organs, inducing glial cells to release ATP, thereby enhancing the activity of GABAergic neurons (Nicole and Pacary, 2020).

Traditional Chinese manual therapy attaches great importance to the attainment of Deqi in clinical practice. Classic theories of Traditional Chinese Medicine (TCM) described Deqi as a state of “pleasant relief upon pressing” (Ma et al., 2014). Previous studies by our research group have indicated that the generation of the Deqi sensation is closely associated with C-fibers (Jiang et al., 2021; Jiang et al., 2024; Jiang et al., 2022). In addition to stimulating sensory nerves in muscles, manual stimulation can also activate C-fibers, and C-fibers can regulate synaptic plasticity in the CNS by activating the Ca^2+^-CaMKII signaling axis. Previous research has demonstrated that mechanical stimulation of tendon organs by manual therapy ameliorated spasticity by activating inhibitory pathways in the CNS (Jiang et al., 2016). Plasticity in synaptic function is manifested as the enhancement or attenuation of signal transmission capacity: upon stimulation, glutamate is released from the presynaptic membrane, which in turn activates NMDAR on the postsynaptic membrane (Klein et al., 2007). That receptor then binds to calmodulin to activate CaMKII, leading to an enhanced postsynaptic response (Jiang et al., 2015). Recent studies have indicated that compression of tendon organs directly triggered the release of GABA, which acted on the corresponding receptors on the postsynaptic membrane of *α*-motor neurons (Yu et al., 2013). A dual inhibitory network is formed via glycinergic interneurons, and signals from compression stimulation are transmitted to the cerebellar cortex through the spinocerebellar tract, thereby alleviating spasticity.

A limitation of the present study is that the pyramidal-tract-lesion model only simulated the spastic symptoms of spastic CP and did not recapitulate the complex pathological etiologies of clinical CP. Future studies can further verify the therapeutic efficacy and underlying mechanisms of manual-therapy intervention in CP models that more closely mimic clinical conditions. The findings of the present study indicated that manual therapy may have regulated neurotransmitter levels in the spinal cord and brain via mechanical stimulation of tendon organs. However, after impairment of C-fiber function by capsaicin in spastic rats, the release of the inhibitory neurotransmitter glycine was reduced, and the improvement in spasticity was attenuated but not completely abolished. This suggested that other afferent pathways may also be involved in mediating the effects of manual therapy, such as Aβ or Aδ fibers.

Additionally, mechanical stimulation may have induced alterations in the local chemical microenvironment, which in turn indirectly activated C-fibers—these aspects warrant further investigation in future research. In the present study, the rats showed no significant recovery trend in soft-tissue tone after the administration of a CaMKII inhibitor. However, the levels of the inhibitory neurotransmitter GABA in the spinal cord and hippocampus increased significantly, and NMDAR levels decreased significantly, which were effects similar to those seen in the MT group. Both the MT group and the CaMKII inhibitor group exhibited better outcomes than did the DMSO solvent (vehicle) group. We hypothesize that intrathecal injection of KN-93 primarily affects the spinal cord directly, whereas manual therapy acts on both the spinal cord and higher-level CNS structures simultaneously.

Although KN-93 administration inhibited CaMKII, manual therapy may have exerted its effects through multiple pathways (both CaMKII-dependent and CaMKII-independent), with CaMKII-independent pathways contributing more significantly to the improvement of muscle tone. CaMKII plays an important role in the effects of Tuina, but it is not the only factor, nor is it the most direct or decisive link in the downstream improvement of muscle tone. The activation or inhibition of CaMKII primarily affects synaptic plasticity and neurotransmitter levels, and the conversion of these changes into behavioral improvements may require the involvement of other conditions or pathways. As a holistic physical stimulation, manual therapy exerts its effects through the integration of multiple targets and pathways, which cannot be fully mimicked by the inhibition of a single molecule.

In summary, this study successfully established a rat model of palsy using anhydrous ethanol-induced pyramidal tract destruction. A key focus of this study was that manual compression of tendon organs effectively alleviated muscle spasticity. The results showed that after tendon organ stimulation by manual therapy, the neurological deficit scores of rats decreased, the morphological structure of the quadriceps femoris on the affected side improved significantly (with reduced inflammatory infiltration), and both muscle tone and muscle spasticity scores decreased significantly. Thus, the mechanism by which manual stimulation of tendon organs alleviates muscle spasticity in CP rats may be associated with the C-fiber-mediated key factor CaMKII. That is, the Deqi effect induced by manual therapy may be transmitted to the spinal cord via C-fibers after impulse generation, thereby modulating synaptic plasticity in spinal inhibitory interneurons. This ultimately promotes the release of more inhibitory neurotransmitter GABA in the spinal cord and brain, suppresses excessive stretch reflexes, and thus reduces muscle tone and relieves spasticity.

## Data Availability

The original contributions presented in the study are included in the article/supplementary material, further inquiries can be directed to the corresponding authors.
